# Correction to: ‘Repercussions of patrilocal residence on mothers’ social support networks among Tsimane forager–farmers’ (2022) by Seabright *et al.*

**DOI:** 10.1098/rstb.2022.0537

**Published:** 2023-04-24

**Authors:** Edmond Seabright, Sarah Alami, Thomas S. Kraft, Helen Davis, Ann E. Caldwell, Paul Hooper, Lisa McAllister, Sarah Mulville, Amanda Veile, Christopher von Rueden, Benjamin Trumble, Jonathan Stieglitz, Michael Gurven, Hillard Kaplan

*Phil. Trans. R. Soc. B* 378, 2021442. (Published online 28 November 2022) (https://doi.org/10.1098/rstb.2021.0442)

A correction is required to the author list and contributions statement for ‘Repercussions of patrilocal residence on mothers’ social support networks among Tsimane forager–farmers’. All of our authors consent to the addition of the author, Amanda Veile. The updated list is as follows:

Edmond Seabright^1,2,†^, Sarah Alami^1,3,†^, Thomas S. Kraft^4^, Helen Davis^5^, Ann E. Caldwell^6^, Paul Hooper^2,7^, Lisa McAllister^8^, Sarah Mulville^9^, Amanda Veile^10^, Christopher von Rueden^11^, Benjamin Trumble^12^, Jonathan Stieglitz^13^, Michael Gurven^3^ and Hillard Kaplan^2^

^1^School of Collective Intelligence, Mohammed 6 Polytechnic University, Ben Guerir 43150, Morocco

^2^Department of Anthropology, University of New Mexico, Albuquerque, NM 87106, USA

^3^Department of Anthropology, University of California Santa Barbara, Santa Barbara, CA 93106, USA

^4^Department of Anthropology, University of Utah, Salt Lake City, UT 84112, USA

^5^Department of Human Evolutionary Biology, Harvard University, Cambridge, MA 01451, USA

^6^Division of Endocrinology, Metabolism and Diabetes, School of Medicine, University of Colorado, Anschutz Medical Campus, Aurora, CO 80217, USA

^7^Economic Science Institute, Chapman University, Orange, CA 92866, USA

^8^Department of Anthropology, Pennsylvania State University, University Park, PA 16802, USA

^9^Institute of Agriculture, University of Tennessee, Knoxville, TN 37996, USA

^10^Department of Anthropology, Purdue University, West Lafayette, IN, USA

^11^Jepson School of Leadership Studies, University of Richmond, Richmond, VA 23173, USA

^12^Center for Evolution and Medicine, Arizona State University, Tempe, AZ 85287-1701, USA

^13^Institute for Advanced Study in Toulouse, Toulouse 1 Capitol University, Toulouse 31080, France

†These authors contributed equally to this study.

Corrected Authors’ contributions:

E.S.: conceptualization, data curation, formal analysis, methodology, visualization, writing—original draft, writing—review and editing; S.A.: conceptualization, data curation, formal analysis, methodology, visualization, writing—original draft, writing—review and editing; T.S.K.: data curation, methodology, writing—review and editing; H.D.: data curation, investigation, writing—review and editing; A.E.C.: investigation, writing—review and editing; P.H.: investigation, writing—review and editing; L.M.: investigation, writing—review and editing; S.M.: investigation, writing—review and editing; A.V.: data curation; C.v.R.: investigation, writing—review and editing; B.T.: investigation, writing—review and editing; J.S.: investigation, writing—review and editing; M.G.: conceptualization, funding acquisition, investigation, methodology, supervision, writing—review and editing; H.K.: conceptualization, funding acquisition, methodology, supervision, writing—review and editing.

All authors gave final approval for publication and agreed to be held accountable for the work performed therein.

This has been corrected on the publisher's website.

